# Retrospective and Prospective Surveillance and Clinical Presentation of Parvovirus B19 in Veneto, Italy, 2024

**DOI:** 10.3390/microorganisms13020430

**Published:** 2025-02-16

**Authors:** Michele Tonon, Stefan-Alexandru Panaite, Davide Gentili, Anna Ferraresso, Filippo Da Re, Debora Ballarin, Francesca Zanella, Maria Teresa Padovan, Michela Pascarella, Sara Mondino, Flavia Riccardo, Anna Teresa Palamara, Nicola Cogo, Marco Milani, Michele Nicoletti, Gloria Pagin, Maria Silvia Varalta, Andrea Cozza, Romina Cazzaro, Francesca Russo, Vincenzo Baldo

**Affiliations:** 1 Regional Directorate of Prevention, Food Safety, Veterinary Public Health, Regione del Veneto, 30123 Venice, Italy; 2 Department of Cardiac Thoracic and Vascular Sciences, and Public Health, University of Padua, 35128 Padua, Italy; 3 Local Health Authority ULSS 8 Berica, 36100 Vicenza, Italy; 4 Department of Infectious Diseases, National Health Institute (Istituto Superiore di Sanità), 00161 Rome, Italy; 5 Regional Directorate for Health Planning, Regione del Veneto, 30125 Venice, Italy

**Keywords:** parvovirus B19, myocarditis, infectious disease surveillance, clinical presentation, epidemiology, comorbidities, fifth disease, erythema infectiosum

## Abstract

The recent increase in parvovirus B19 (B19V) infections across Europe has underscored the need for enhanced surveillance. In Italy, B19V surveillance is not mandated nationally. This ambispective (retrospective and prospective) study aimed to assess the epidemiological and clinical characteristics of the 2024 apparent surge in the Veneto Region by implementing mandatory disease notification starting in May 2024 and collecting clinical data on confirmed cases. During the surveillance period, 3156 B19V cases were reported, with 2.9% (93/3156) requiring hospitalisation (95% CI 2.4–3.5%). Children under 1 year of age exhibited the highest hospitalisation rate (29.0%), followed by adults aged 18–40 (17.5%). Infections disproportionately affected children aged 1–5 and 6–11, and the Granger causality test showed that an increase in cases among the 1–5-year-olds was predictive of subsequent rises in other age groups (*p* = 0.0086). Severe complications, including myocarditis, pericarditis, and miscarriage, were observed, and one death occurred following cardiac and neurological complications in a patient without prior risk factors. The presence of concurrent pathologies, particularly haematological disorders, was associated with increased disease severity. These findings highlight the importance of early warning when cases begin rising among preschool-aged children and underscore the need for improved B19V awareness, particularly in high-risk populations. Future efforts should evaluate the feasibility of implementing a systematic, potentially EU-wide, surveillance for B19V.

## 1. Introduction

Parvovirus B19 (B19V) is a globally distributed single-stranded DNA virus from the *Parvoviridae* family that commonly causes mild, self-limiting infections in children, presenting as erythema infectiosum (“slapped cheek” rash). In adults and high-risk populations, signs and symptoms may vary and often include arthralgia and anemia. Notably, up to 25% of infections in both children and adults may be asymptomatic. Subclinical cases with cold-like symptoms are also reported but rarely attributed to B19V [1,2,3]. Hospitalisation data for B19V infections are notably scarce, as most studies focus on epidemiological patterns or specific complications. Even comprehensive reviews seldom include hospitalisation rates [1,2]. A recent study in France reported a 1.8% hospitalisation rate among children younger than 15 years visiting emergency departments during the 2023–2024 outbreak, marking an increase from 1.2% in previous seasons [4].

Specific populations, including individuals with red blood cell deficiencies, immunocompromised patients, and pregnant women face elevated risks and complications. Individuals with red blood cell deficiencies or other hematological disorders may develop aplastic crises or congestive heart failure, particularly in pediatric cases. Immunocompromised patients can progress to chronic B19V infection, often presenting with pure red cell aplasia without the classic rash and joint involvement [2]. In pregnant women, the infection can be transmitted to the fetus (17–35% risk), typically 1 to 3 weeks after maternal infection, potentially leading to severe anemia, nonimmune hydrops fetalis, and intrauterine fetal death, especially when maternal infection is acquired between 9 and 16 weeks of gestation [5].

Beyond these well-established complications, B19V has also been associated with other conditions, including hepatitis, myocarditis, autoimmune disorders, and diverse neurological presentations [2,3,6,7]. Although these and other associations have long been considered rare, recent studies suggest underreporting due to lack of testing and awareness or indicate a stronger-than-expected association [8,9]. A recent systematic review and meta-analysis reported a 23.7% (95% CI: 18.7–29.5%) prevalence of B19V infection in myocarditis, suggesting a possible yet uncertain causal relationship. Indeed, a similar prevalence of the virus was found in endomyocardial biopsies of healthy subjects and of those with myocarditis and dilated cardiomyopathy. It has been suggested that B19V may play a role in conjunction with other viruses, or pathogenesis may depend on viral load or replication activity [8].

Generally, individuals develop lifelong immunity following infection, and seroconversion rates increase globally with age, varying from 2% to 20% in children under 5 years, 15% to 40% in those aged 5–18 years, to 40–80% in adults [3]. During the COVID-19 pandemic, B19V circulation sharply declined, potentially reducing population immunity and increasing the risk of subsequent rebound outbreaks [10,11]. In April 2024, the European Centre for Disease Prevention and Control (ECDC) reported an increase in B19V infection notifications across several EU/EEA countries towards the end of 2023 and during 2024 [12]. In Italy, routine surveillance for B19V has not been legally mandated, and published data derive from plasma donation screening rather than systematic epidemiological reporting [13,14].

The objective of this study is, first and foremost, to assess the epidemiological situation of B19V infections in the Veneto Region, Italy, to determine whether the recent European resurgence has affected the region.

A secondary aim is to collect clinical data in both low- and high-risk populations, focusing on hospitalised cases, addressing the notable scarcity of systematic data on B19V-related hospitalisations.

Finally, the study seeks to contribute to the limited literature on rare B19V-related conditions, such as myocarditis.

## 2. Materials and Methods

### 2.1. Study Design

In response to the ECDC Report, an observational study was conducted to assess the epidemiology of B19V infection in the Veneto Region, Italy, from 1 January 2024, to 4 August 2024 (weeks 1–31). The study represents a retrospective cohort design for data collected before the introduction of mandatory notification (1 January to 2 May 2024) and an enhanced prospective surveillance design for data collected thereafter (3 May to 4 August 2024).

### 2.2. Case Definition

The cases reported in the study include probable and confirmed cases. Probable cases were defined as those presenting with erythema infectiosum, characterised by the hallmark slapped cheek sign, and/or an epidemiological link to a confirmed case, excluding other probable causes.

The diagnostic approach for confirming B19V infection, in accordance with ECDC criteria, includes quantitative polymerase chain reaction (qPCR) to detect viral DNA and serological testing for IgM and IgG antibodies, with rising IgM titers indicating a recent infection [14]. Serological testing was conducted using a chemiluminescent immunoassay (CLIA) to detect IgM and IgG antibodies in serum or plasma samples (LIAISON^®^ Parvovirus B19 assays panel, DiaSorin Italia S.p.A, Saluggia, Italy), while nucleic acid testing involved quantitative real-time PCR (Parvovirus B19 ELITe MGB^®^ Kit, ELITechGroup S.p.A, Torino, Italy) performed on whole blood or amniotic fluid samples to detect viral DNA. Diagnostic procedures for pregnant women were conducted in accordance with the national AMCLI (Associazione Microbiologi Clinici Italiani) guidelines, ensuring that testing and interpretation adhered to the latest evidence-based standards [15]. The selection of tests was individualised by attending physicians based on clinical presentation and specific patient populations. Molecular testing (qPCR) was performed in all hospitalised patients, immunocompromised individuals, pregnant women, or when specifically requested by the prescribing physician. Serological testing was conducted in all hospitalised patients (alongside qPCR) and in any case where requested by the physician.

### 2.3. Data Collection

The Veneto Region issued a communication on 3 May 2024 (week 18), addressed to all Local Health Authorities (LHAs), Hospital Organisations, and the Veneto Institute of Oncology. The communication required the following actions:

- mandatory reporting of all cases of B19V infection;
- epidemiological investigation of all reported cases to identify contacts with individuals at high risk;
- provision of diagnostic testing free of charge for all high-risk contacts associated with probable or confirmed cases.

Clinicians were also requested to provide retrospective data on confirmed cases diagnosed from 1 January 2024 onwards. Information regarding the risks associated with B19V infection in pregnant women was disseminated. The directive also recommended collaboration with relevant hospital departments and clinicians, including Obstetrics and Gynecology, Pediatrics, Microbiology, Infectious Diseases, General Practitioners, and Pediatricians, to ensure effective implementation of the outlined measures.

Physicians reported positive cases by submitting documentation to their respective Public Health Office, part of the Local Health Authorities (LHAs). Public Health staff conducted epidemiological investigations and forwarded the information to the regional and national authorities through the Regional Infectious Diseases Notification Platform, SIRMI. SIRMI is integrated with the National Disease Surveillance Platform, PREMAL.

The regional authority, under the national regulation, accessed data from the infectious disease surveillance platform and conducted an analysis of disease trends for public health purposes. Data collected included patient demographics, date of symptom onset as reported by the patient, date of notification, clinical presentation, hospitalisation status, and any comorbidities.

### 2.4. Data Organisation and Analysis

Anonymised data were organised and stored using Google Sheets (Google LLC, Mountain View, CA, USA) for preliminary management and presentation. The information was categorised by age groups, symptom onset dates, notification dates, hospitalisation status, and comorbidities.

Data analysis was performed using R version 4.0.3 (2020-10-10, R Core Team, Vienna, Austria) in conjunction with RStudio (version 2021.09.1 Build 372, RStudio PBC, Boston, MA, USA). Descriptive statistics were used to summarise the distribution of cases and hospitalisation rates across different age groups. Confidence intervals were calculated to estimate the proportion of severe cases requiring hospitalisation. A chi-square test with Bonferroni correction was employed to assess whether the likelihood of hospitalisation differed across various age groups.

The clinical presentations of hospitalised individuals were analysed both collectively and by comorbidity. To support the discussion of reported symptoms and comorbidities, the most frequent clinical presentations were categorised into eight broad areas: generic, dermatologic, rheumatologic, cardiac, hematologic, neurologic, obstetric, and other. Briefly, “generic” included fever, asthenia, and loss of appetite; “dermatologic” encompassed exanthema or rash; “rheumatologic” involved arthralgia and myalgia; “cardiac” included myocarditis and pericarditis; “hematologic” covered anaemia and various cytopenias; “neurologic” included ataxia and suspected encephalitis; “obstetric” involved miscarriage; and “other” comprised less frequent or miscellaneous symptoms. Full details on the classification process and the complete symptom list are provided in Supplementary Materials File S1.

Finally, Granger causality analysis was employed to assess whether the temporal trends of cases in the 1–5 years and 6–11 years age groups, characterised by a lower seroprevalence, influenced trends and informed the prediction of future cases in other age groups, thereby suggesting a potential causal relationship. A 3-day lag was applied in the Granger causality test to account for potential delays in transmission dynamics, considering that the onset of the disease within the first 3 days following exposure is not supported by the available evidence.

Finally, a binary logistic regression model was applied to evaluate the impact of the independent variables (age, sex, and presence of comorbidities) on the dependent variable, hospitalisation. The Hosmer–Lemeshow test was conducted to assess the model’s goodness of fit.

## 3. Results

### 3.1. Epidemiological Trends

Preliminary data on B19V surveillance in Veneto for 2024, as of week 31 (29 July–4 August), indicate an increase in cases beginning primarily from epidemiological week 17 (22–28 April), exceeding the typical seasonal trend of approximately 50 cases observed during this week. This was followed by a peak in week 21 (20–26 May), with a subsequent steady decline in the following weeks, reaching the lowest number of cases in week 31. During the surveillance period, 3156 B19V cases were reported to the Local Health Authorities (LHAs), while laboratories identified 684 NAAT positive cases and detected 3061 cases positive for both IgM and IgG antibodies, resulting in an almost one-to-one match between case notifications and laboratory confirmations.

**Figure 1:**
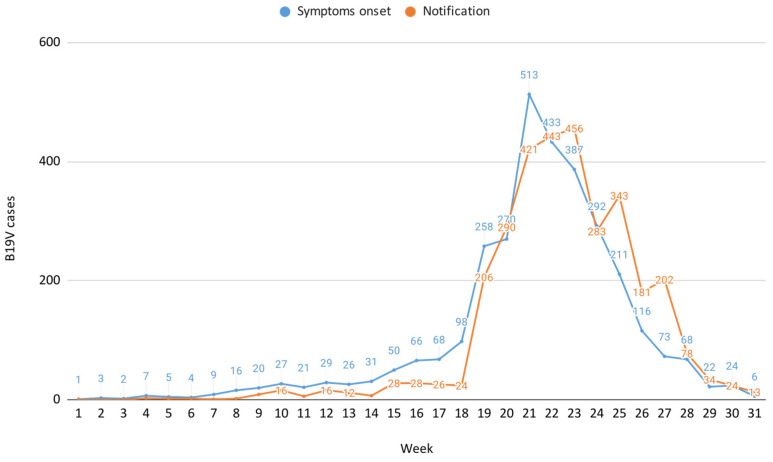
B19V case distribution by symptom onset and notification, weeks 1–31, 2024.

The B19V case distribution by symptom onset and notification, weeks 1–31, 2024 is reported in Figure 1, which also illustrates a notification delay of approximately one week compared to the onset of symptoms, consistent with reporting at the time of rash appearance.

### 3.2. Demographic Distribution, Influence on Transmission, and Hospitalisation Rates

**Figure 2:**
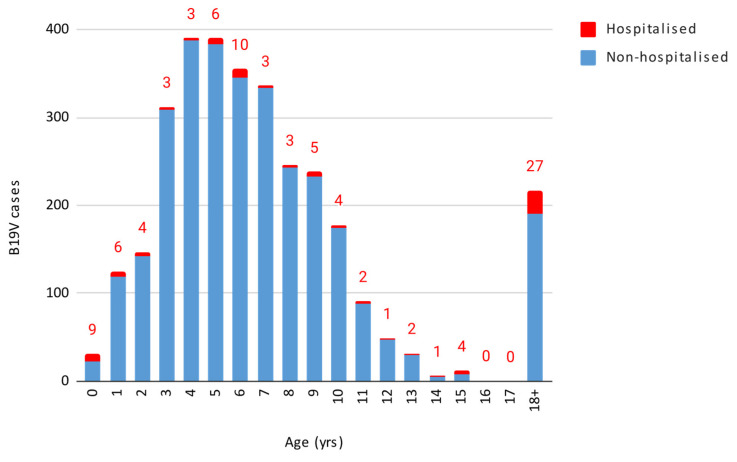
Number of B19V cases and hospitalisation by age.

Out of the 3156 cases, 1619 were females and 1537 were males, without a statistically significant difference. The infection predominantly impacted children aged 1–5 and 6–11 years, with both age groups showing a peak in week 21, consistent with the overall data. These age groups overlap with the typical age of nursery and elementary school students. Figure 2 displays the number of B19V cases and hospitalisations by age.

The results were as follows when performing the Granger causality test to determine whether the age groups with the highest number of cases affected the incidence in the other age groups, with a 3-day lag. The 1–5 years age group had a significant influence both on the incidence of cases in all other age groups combined (*p*-value = 0.0086) and individually on the 6–11 years (*p*-value = 0.000499) and 18–40 years age groups (*p*-value = 0.0146). Conversely, the 6–11 years age group did not show a statistically significant influence on all other age groups combined or on most other age groups when considered individually, except for a potential effect on those under 1 year of age (*p*-value = 0.0292).

Out of 3156 cases, 93 (2.9%, 95% CI 2.4–3.5%) required hospitalisation, with varying rates across different age groups (Table 1).

The highest hospitalisation rate (29%) was observed in children under 1 year of age, although this includes infants hospitalised for postnatal monitoring. The 18–40 age group exhibited a hospitalisation rate of 17.5%, the 12–17 age group 8%, and the over-40 age group 5.5%. The chi-squared test revealed that children under 1 year of age and adults aged 18–40 years had significantly higher hospitalisation rates compared to other age groups (*p* < 2.2 × 10^−16^). The findings remained significant after applying the Bonferroni correction for multiple comparisons, (adjusted α = 0.0033).

The logistic regression model is presented in Table 2.

The binary logistic regression model highlights that the independent variables age and presence of comorbidities significantly influence the likelihood of hospitalization. The Hosmer–Lemeshow goodness-of-fit test indicated an adequate fit for the logistic regression model (χ^2^ = 13.36, *p* = 0.0999).

### 3.3. Clinical Presentation

Clinical presentation data were available for 50 of the 93 hospitalised individuals. The most common signs and symptoms at admission were fever (18/50 cases, 36.0%), anemia (11/50 cases, 22.0%), skin rash (7/50 cases, 14.0%), and arthralgia or myalgia (7/50 cases, 14.0%).

Examining the severity of clinical presentations, two women were admitted for miscarriage during the second trimester of pregnancy. Two cases developed myocarditis, while another case presented with pericarditis. Additionally, two more cases were admitted for suspected myocarditis. One particularly severe case involved a patient admitted with cardiorespiratory arrest, acute renal failure, and anoxic-ischemic brain injury with cerebral oedema, who ultimately died. Another case developed Henoch-Schönlein purpura with cutaneous, articular, and gastrointestinal involvement. Neurological presentations included one case of febrile seizures, one case of acute-onset ataxia, and one case of severe headache with speech impairment, which was diagnosed as possible encephalitis. Hematological presentations included one case of hemolytic crisis, one case of vaso-occlusive crisis, one case of cytopenia, one case of medullary aplasia, one case of leukopenia, and two cases of leuko-thrombocytopenia, along with one case of an unspecified alteration of the hemogram. Other nonspecific presentations included asthenia (2 cases), reduced appetite (1 case), and low-grade fever below 38 °C (1 case). Additional dermatological presentations included urticaria (2 cases).

### 3.4. Comorbidities and Pregnancy in Hospitalised Cases

Comorbidities were present in 20 out of 93 (21.5%) hospitalised cases. Hematological diseases were the most common comorbidity, accounting for 60% (12/20) of cases. These included spherocytosis (4 cases), beta thalassemia minor (2 cases), sickle cell disease (2 cases), glucose-6-phosphate dehydrogenase deficiency, leukemia, heterozygous HbS/HbC, and an unspecified hemoglobinopathy. Infectious diseases were the next most common comorbidity, representing 25% (5/20) of cases, including Epstein–Barr virus (n = 2), tuberculosis (n = 1), enterovirus detected in cerebrospinal fluid (n = 1), and an unspecified bacterial infection. Other comorbidities included an unspecified malignancy, multiple sclerosis, and an eating disorder.

Clinical presentation was available for 13 out of the 20 hospitalised patients with comorbidities. The distribution of symptoms among hospitalised individuals varied depending on comorbidity status. In patients without comorbidities, generic symptoms were the most prevalent (48.6%, 18/37 cases), followed by hematologic (32.4%, 12/37 cases), dermatologic (24.3%, 9/37 cases), rheumatologic (16.2%, 6/37 cases), and neurologic (10.8%, 4/37 cases) symptoms. Additionally, 5.4% (2/37 cases) reported miscarriage and 37.8% (14/37 cases) experienced “other” symptoms. Hematological disorders were most frequently associated with hematologic symptoms (77.8%, 7/9 cases) and less commonly with generic (11.1%, 1/9 cases) and rheumatologic (11.1%, 1/9 cases) symptoms. Infectious comorbidities were primarily linked to generic symptoms (50%, 1/2 cases) and “other” symptoms (50%, 1/2 cases). Other comorbidities were associated with generic symptoms (50%, 1/2 cases) and cardiac and “other” symptoms (50%, 1/2 cases each).

It should be highlighted that all cases of miscarriage (n = 2), myocarditis (n = 2), suspected myocarditis (n = 2), Henoch-Schönlein purpura (n = 1), febrile seizures (n = 1), ataxia (n = 1), and possible encephalitis (n = 1) occurred in individuals with no reported comorbidities. The case presenting with pericarditis occurred in a patient with a documented eating disorder. The unique case presenting with cardiorespiratory arrest, acute renal failure, and anoxic-ischemic brain injury accompanied by cerebral oedema, was also in a patient with no reported comorbidities. Anaemia was observed in both patients with haematological disorders (5 out of 12 cases with reported data) and those without comorbidities (6 out of 37 cases with reported data). Cases of hemolytic crisis (n = 1) and vaso-occlusive crisis (n = 1) were reported exclusively in individuals with hematologic comorbidities, whereas other hematologic presentations, such as cytopenia (n = 1), medullary aplasia (n = 1), leukopenia (n = 1), and leuko-thrombocytopenia (n = 2) occurred in individuals with no reported comorbidities. No comorbidities were reported in individuals presenting with a skin rash.

Pregnancy was reported in 4.3% (4/93) of hospitalised cases. Three of these women had no concomitant diseases or symptoms. One woman, who had beta thalassemia minor, required blood transfusions due to anaemia.

## 4. Discussion

This study provides valuable insights into the epidemiological patterns, clinical characteristics, and impact of comorbidities in the 2024 parvovirus B19 apparent surge in the Veneto Region. The implications of these findings are discussed in the context of existing literature, considering the potential role of comorbidities and co-infections in disease severity, as well as outlining limitations and future directions for B19V surveillance and research.

Implementing mandatory reporting for B19V significantly enhanced prospective data collection, enabling a comprehensive analysis of its epidemiological trajectory and insights into unusual severe manifestations of the disease. Continued surveillance in this setting could facilitate rapid detection and response to future outbreaks while contributing valuable data to the national and international public health framework. Consequently, the Veneto Region will maintain ongoing monitoring. Additionally, implementing genotypic surveillance could enable the detection of specific B19V genotypes during outbreaks.

This study suggests a possible B19V circulation during late winter and spring, followed by a decline in cases as summer approaches, likely reflecting the expected seasonality of this virus [1,2,3,11]. Additionally, the study captures the decline phase of the B19V epidemiological curve, providing precise epidemiological insights into the local progression of the infection trend. Surveillance data showed that cases continued to occur until June–July, suggesting prolonged onward transmission even outside its typical seasonal window. This underscores the importance of understanding local factors, such as population immunity gaps or environmental conditions, that may influence transmission dynamics. The reporting system operated with a one-week delay relative to the onset of symptoms, which aligns with a typically delayed healthcare-seeking behaviour, whereby patients do not immediately consult general practitioners or specialists for testing and probably wait until the development of a cutaneous rash.

The analysis of age-related transmission patterns indicates that younger children, particularly those aged 1–5 years, played a significant role in the spread of the virus, possibly triggering the circulation and amplification of transmission in other age groups. This is consistent with prior research, which identifies young children as the most susceptible to and infected with B19V [3]. Settings such as daycare centres and preschools likely act as key transmission hubs due to the close contact and interaction among children, coupled with the high susceptibility of this age group to respiratory and exanthematous infections [16,17]. However, to our knowledge this study is the first to demonstrate a direct impact of infections in the 1–5 years age group on other age groups. It is important to emphasize, however, that the Granger causality analysis used to assess this relationship identifies temporal associations rather than definitive causative pathways. Further research is needed to validate these findings and explore the biological mechanisms underpinning this pattern. These findings suggest that public health interventions targeting the 1–5 years age group could significantly impact infection control, both in the 1–5 age group and in the remaining age groups. Such interventions may include enhanced infection control measures in early childhood education environments and targeted public health messaging for parents [18]. Additionally, targeted monitoring of cases in the 1–5 years age group could serve as an early warning for local healthcare authorities, facilitating improved preparedness and response to the spread of infection to other age cohorts in subsequent weeks.

Although severe and life-threatening disease as a consequence of infection was relatively rare overall, hospitalisation rates were notably high in certain age groups, including the <1 year cohort, which had a hospitalisation rate of 29%, and the 18–40 years cohort, with a rate of 17.5%. These rates are considerably higher than the overall 2.9% hospitalisation rate and exceed those reported in a recent study, which reported a 1.8% hospitalisation rate among children under 15 years attending the emergency department [4]. Among hospitalised cases, the clinical presentation varied, with fever, anemia, skin rash, and arthralgia or myalgia being the most prevalent symptoms. Notably, some cases exhibited severe complications including myocarditis, pericarditis, and severe neurological conditions such as possible encephalitis, which have been increasingly linked to B19V [1,8,19,20,21]. The occurrence of myocarditis is particularly notable, as it underscores the cardiotropic nature of B19V. A recent study by Poeta et al. documented 65 paediatric myocarditis cases in Italy during the same timeframe, with nearly half testing positive for B19V [21]. These findings suggest that B19V may act as a primary etiological agent in pediatric myocarditis, contributing to severe clinical outcomes, including heart failure and death. In addressing potential confounding factors, it is important to consider the role of antibiotic usage during the prenatal and postnatal periods. Recent studies have identified a significant association between early-life antibiotic exposure and an increased risk of neuropsychiatric disorders in children. Specifically, prenatal antibiotic use was associated with a 7% increased risk of such disorders, while postnatal exposure was linked to a 5% increase. Moreover, a synergistic effect was observed when both exposures were present, with a 12% increased risk [22]. These findings underscore the importance of careful benefit–risk assessment when prescribing antibiotics during early life. While our dataset does not allow us to assess antibiotic usage as a confounding factor in this study, this issue remains an important area for future exploration. The occurrence of two miscarriages underscores the well-documented risks of B19V in pregnancy [23,24]. This aligns with recent findings by Russcher et al., who reported adverse outcomes, including perinatal death, termination of pregnancy, persistent hydrops, or severe cerebral anomalies, in 36% of fetuses who required intrauterine transfusions during the 2023–2024 parvovirus B19 epidemic in northwestern Europe [25]. Some authors have even suggested evaluating the introduction of universal serological testing for B19V in pregnant women during epidemic periods, to enable early infection management and potentially reduce adverse fetal outcomes [26]. These findings highlight the need to raise awareness among clinicians about the potential for severe complications in a disease typically considered to be mild. Prompt recognition is critical for appropriate follow-up and treatment strategies, especially in pregnant women, individuals with cardiac complications, and those with anemia [27,28]. Moreover, pregnant women with comorbidities, such as immunosuppression or pre-existing hematological disorders, may be at heightened risk for adverse outcomes due to overlapping vulnerabilities. Likewise, immunocompromised patients, including those on immunosuppressive therapies or with immune-modulating conditions, face a particularly high risk of persistent infection which, when coupled with additional comorbidities, can further exacerbate clinical manifestations and increase morbidity.

In this study, the preliminary analysis of hospitalisation drivers further indicates that age and comorbidities significantly predict the likelihood of hospital admission, as demonstrated by a logistic regression model. These findings reinforce the disproportionate burden observed in the <1 year and 18–40 years cohorts, underscoring the need for heightened clinical vigilance and targeted surveillance in these age groups.

Ultimately, the presence of concurrent pathologies in a significant proportion of hospitalised cases (22%) suggests that these comorbidities may exacerbate the severity of the disease, and vice versa. The high prevalence of concurrent haematological disorders in hospitalised cases, including spherocytosis and beta-thalassemia minor, highlights the interaction between B19V and underlying conditions. This is consistent with findings from other outbreaks, where individuals with haematological disorders were disproportionately affected by severe B19V complications [3,4]. Additionally, co-infections with pathogens such as Epstein–Barr virus, enteroviruses, and tuberculosis were present in 25% (5/20) of cases. Literature also suggests that B19V co-infections with other viruses, such as influenza or Epstein–Barr virus, may exacerbate disease severity and further complicate clinical outcomes [29,30,31]. These co-infections likely contribute to more severe clinical presentations, as the combined immune response could exacerbate systemic inflammation or hinder viral clearance. This underscores the need for clinicians to remain vigilant for concurrent infections when managing severe B19V cases.

In conclusion, our findings underscore the importance of targeted public health interventions, particularly among high-risk groups, and highlight areas where enhanced surveillance could strengthen outbreak detection and management, aligning with recent recommendations from the Italian Ministry of Health [30]. Specifically, the Ministry has recommended that all Regions and Autonomous Provinces increase physician awareness of B19V, communicate risks to vulnerable groups (such as pregnant women, immunocompromised individuals, and patients with chronic blood disorders), and conduct multidisciplinary analyses of historical data to track transmission trends and patterns [32,33]. Future research should focus on understanding B19V’s interactions with co-infections and assessing the benefits of a national surveillance framework. Integrating these findings will help refine public health responses and improve clinical outcomes in subsequent epidemic surges.

This study has some limitations. First, variations in thresholds for hospital admission across different facilities may have created reporting discrepancies, potentially influencing the observed rates of severe disease and hospitalisations. Additionally, the reliance on retrospective data collection introduces the potential for biases, such as recall bias or incomplete records, which could have impacted the accuracy and completeness of the dataset. Misclassification of cases is another possibility, given the lack of systematic confirmatory testing and the reliance on physician-reported diagnoses, particularly in cases where only serological testing was performed. Most of the reported cases lacked microbiological data, as national reporting of B19V is not mandatory and no structured data collection form is in place. Instead, clinical data reporting relies on voluntary contributions from healthcare providers alongside the mandatory notification, which can result in incomplete datasets. This lack of obligation to report limits the systematic tracking of severe cases and hinders the ability to obtain comprehensive epidemiological and clinical insights into B19V infections. Additionally, it was impossible to cross-reference patient laboratory data with notification records due to regulations governing the processing of personal data. Furthermore, the request for retrospective data has not led to the collection of substantial cases before the request for mandatory notification. This may be attributed to a lower epidemiological presence of B19V in the region and potential underdiagnosis due to low awareness and insufficient testing, alongside underreporting of historical cases. This inference is supported by the limited but existent national data on B19V-positive plasma units intended for fractionation.

Another key limitation is the uncertainty regarding the nature of the reported comorbidities. It is unclear whether these comorbidities represent longstanding conditions, as the term suggests, or newly diagnosed concurrent diseases, such as infectious conditions like Epstein–Barr virus, enterovirus, or tuberculosis, identified at the time of hospitalisation. Additionally, clinical presentation data were unavailable for 43 out of the 93 hospitalised cases (46%), which weakens the robustness of the findings regarding hospitalisation rates and severity patterns. Some patients, such as newborns and pregnant women, may have been hospitalised for testing or reasons unrelated to the infection, potentially inflating or distorting these figures. As a result, we are unable to conduct sub-analyses on important variables, potentially missing trends or risk factors for severe disease. Strengthening data collection protocols—such as ensuring consistent documentation of admission reasons and clinical presentations—would help mitigate this limitation in future investigations. We also acknowledge that antibiotic usage during both fetal and postnatal life has been associated with an increased risk of childhood neuropsychiatric disorders. This factor was not accounted for in this dataset and warrants further study.

It should also be noted that the claim regarding the 1–5-year-old age group significantly influencing infection in other age groups based on Granger causality analysis is preliminary. The underlying assumptions and limitations of this statistical method were not fully addressed in this study. Further research will be necessary to confirm these findings.

## 5. Conclusions

This study contributes to the broader understanding of the disease’s transmission dynamics, clinical manifestations, and the impact of comorbidities. Our findings underscore the role of younger children, particularly those aged 1–5 years, as a potential driver of transmission, highlighting the need for targeted public health interventions in early childhood settings, such as enhanced infection control measures and parental awareness campaigns.

The study also emphasizes the clinical impact of severe complications, including myocarditis and adverse pregnancy outcomes, which reinforce the need for heightened clinical vigilance, especially in high-risk groups such as pregnant women, individuals with hematological disorders, and immunocompromised patients. The findings provide valuable insights for public health authorities and clinicians, offering a basis for improved prevention and treatment strategies in future epidemic surges.

The feasibility of implementing a mandatory systematic and potentially EU-wide surveillance for B19V, with active international communication and collaboration, should be explored. Such surveillance could allow for more accurate tracking of epidemiological trends, timely detection of outbreaks, and improved response coordination. At the national level, adopting standardized data collection protocols and integrating laboratory and clinical databases would ensure data consistency, enhance interoperability, and strengthen the effectiveness of public health surveillance systems. Additionally, integrating B19V genomic surveillance could provide additional valuable insights on viral diversity and pathogenicity, further guiding targeted public health interventions. Specifically, genotypic analysis during outbreaks could help identify emerging strains with pathogenic potential, enabling tailored strategies to mitigate severe outcomes.

Future research should focus on confirming the preliminary findings on age-related transmission dynamics, understanding the mechanisms linking B19V to severe complications such as myocarditis, and exploring the impact of co-infections and comorbidities on disease severity. Strengthening these knowledge gaps will refine public health responses and enhance clinical outcomes during subsequent outbreaks.

## Figures and Tables

**Table 1:** B19V cases by age group and hospitalisation (January–August 2024). The *p*-values presented in this table have been adjusted using the Bonferroni correction method to account for multiple comparisons.

Age Group	B19V Cases, n	Hospitalised, n (%)	Adjusted *p*-Value	Hospitalization Contribution by Individual Age Group (%)
<1	31	9 (29.0)	1.312838 × 10^−15^ ***	9.7
1–5	1364	22 (1.6)	5.366575 × 10^−2^	23.7
6–11	1444	27 (1.9)	2.006811 × 10^−1^	29.0
12–17	100	8 (8.0)	2.466041 × 10^−1^	8.6
18–40	126	22 (17.5)	2.211724 × 10^−17^ ***	23.7
>40	91	5 (5.5)	9.681672 × 10^−1^	5.4
**Total**	**3156**	**93 (2.9)**	**-**	**100.0**

*** indicates *p* < 0.001.

**Table 2:** Binary logistic regression model assessing the influence of age, gender, and previous clinical condition on the hospitalisation status among B19V cases.

Number of obs = 3156					
LR chi^2^ (3) = 61.67					
Prob > chi^2^ = 0.0000					
Log likelihood = –388.62856			Pseudo R^2^ = 0.0730		
Hospitalisation	Odds Ratio	z	*p* > |z|	(95% Conf. Interval)	
**Age**	1.040512	5.51	<0.001	1.025917	1.055313
**Sex**	1.271733	1.07	0.284	0.8195206	1.973477
**Previous** **condition**	69.34686	5.88	<0.001	16.87567	284.9656

## Data Availability

The original contributions presented in this study are included in the article/Supplementary Materials. Further inquiries can be directed to the corresponding author.

## Supplementary Materials

The following supporting information can be downloaded at: https://www.mdpi.com/article/10.3390/microorganisms13020430/s1, File S1: Parvovirus B19 signs/symptoms categorisation.
