# Supplementary material for: Retrospective and Prospective Surveillance and Clinical Presentation of Parvovirus B19 in Veneto, Italy, 2024
Source: Microorganisms. 2025 Feb 16;13(2):430. doi: 10.3390/microorganisms13020430 (PMC11858032; doi:10.3390/microorganisms13020430)
Supplement: Supplementary file 1 [file microorganisms-13-00430-s001.zip › microorganisms-3400144-supplementary.pdf]

S1: Parvovirus B19 signs/symptoms categorisation

To facilitate data analysis and interpretation, symptoms were independently categorised by two expert physicians into broad clinical areas in a blinded manner. This data-driven organisation, developed based on the collected data rather than predefined categories, provides a clear understanding of symptom prevalence and aids in cross-referencing with comorbidities, as discussed in the dedicated section.

Clinical presentations were categorised as follows:

- **Generic symptoms**: fever, asthenia, loss of appetite.
- **Dermatologic**: exanthema or rash, pruritus, Henoch-Schönlein purpura without gastrointestinal involvement.
- **Rheumatologic**: arthralgia, myalgia, Henoch-Schönlein purpura.
- Cardiac: myocarditis, suspected myocarditis, pericarditis, cardiorespiratory arrest.
- **Haematologic**: anaemia, haemolytic crisis, vaso-occlusive crises, cytopenia, bone marrow aplasia, leukopenia, and leuko-thrombocytopenia.
- **Neurologic**: anoxic-ischaemic brain injury with cerebral oedema, febrile convulsions, ataxia, and suspected encephalitis.
- **Obstetric**: miscarriage.
- **Other**: rectorrhagia, acute renal failure, microhematuria, cough accompanied by dehydration, right supraclavicular lymphadenopathy and mild splenomegaly, dependent oedema, pain crises, anorexia, Henoch-Schönlein purpura with gastrointestinal involvement, weight loss, jaundice, hyposthenia of the lower limbs, acute dehydration, generalised pain.
